# Multi-Role Surface Modification of Single-Crystalline Nickel-Rich Lithium Nickel Cobalt Manganese Oxides Cathodes with WO_3_ to Improve Performance for Lithium-Ion Batteries

**DOI:** 10.3390/nano12081324

**Published:** 2022-04-12

**Authors:** Limin Ou, Shengheng Nong, Ruoxi Yang, Yaoying Li, Jinrong Tao, Pan Zhang, Haifu Huang, Xianqing Liang, Zhiqiang Lan, Haizhen Liu, Dan Huang, Jin Guo, Wenzheng Zhou

**Affiliations:** Guangxi Novel Battery Materials Research Center of Engineering Technology, Guangxi Colleges and Universities Key Laboratory of Novel Energy Materials and Related Technology, Guangxi Key Laboratory of Processing for Non-ferrous Metallic and Featured Materials, School of Physical Science and Technology, Guangxi University, Nanning 530004, China; 1907301067@st.gxu.edu.cn (L.O.); 2007301111@st.gxu.edu.cn (S.N.); 2107301160@st.gxu.edu.cn (R.Y.); 2107301062@st.gxu.edu.cn (Y.L.); 1812110135@st.gxu.edu.cn (J.T.); 1812110145@st.gxu.edu.cn (P.Z.); huanghf@gxu.edu.cn (H.H.); lxq@gxu.edu.cn (X.L.); l_zq1100@163.com (Z.L.); liuhz@gxu.edu.cn (H.L.); danhuang@gxu.edu.cn (D.H.); guojin@gxu.edu.cn (J.G.)

**Keywords:** nickel-rich NCM, single-crystalline, surface modification, WO_3_

## Abstract

Compared with the polycrystalline system, the single-crystalline ternary cathode material has better cycle stability because the only primary particles without grain boundaries effectively alleviate the formation of micro/nanocracks and retain better structural integrity. Therefore, it has received extensive research attention. There is no consistent result whether tungsten oxide acts as doping and/or coating from the surface modification of the polycrystalline system. Meanwhile, there is no report on the surface modification of the single-crystalline system by tungsten oxide. In this paper, multirole surface modification of single-crystalline nickel-rich ternary cathode material LiNi_0.6_Co_0.2_Mn_0.2_O_2_ by WO_3_ is studied by a simple method of adding WO_3_ followed by calcination. The results show that with the change in the amount of WO_3_ added, single-crystalline nickel-rich ternary cathode material can be separately doped, separately coated, and both doped and coated. Either doping or coating effectively enhances the structural stability, reduces the polarization of the material, and improves the lithium-ion diffusion kinetics, thus improving the cycle stability and rate performance of the battery. Interestingly, both doping and coating (for SC-NCM622-0.5%WO_3_) do not show a more excellent synergistic effect, while the single coating (for SC-NCM622-1.0%WO_3_) after eliminating the rock-salt phase layer performs the most excellent modification effect.

## 1. Introduction

Lithium-ion batteries (LIBs) are widely applied in portable consumer electronics, pure electric vehicles (PEV) and hybrid electric vehicles (HEV) because of their high energy density, low self-discharge, and green environmental protection [1,2]. One of the challenges of large-scale application of state-of-the-art LIBs in the electromobility consumer market is the further increase in mass-energy density (Wh·kg^−1^) and volume-energy density (Wh·L^−1^), which results from the demand of long driving range [2,3]. For cathode materials, layered lithium nickel cobalt manganese oxides (NCM), Li[Ni_x_Co_y_Mn_1−x−y_]O_2_ (0 < x < 1, 0 < y < 1, 0 < 1 – x − y < 1), are more prosperous due to their outstanding properties such as more stable structure, better cycle stability, higher specific capacity and lower cost compared with LiNiO_2_, LiCoO_2_ and LiMnO_2_ (LiMO_2_, M = Ni, Co, Mn) [4,5,6]. In NCM composites, the content of the Ni element dominates the capacity and the Mn element stabilizes the structure. Generally, NCM composite with x ≥ 0.6 is called high-Ni (Ni-rich) cathode material. The greater the Ni content is, the higher the specific capacity is [7,8].

However, accompanied with the increase in Ni content, some problems appear, such as poor cycle stability, low initial coulombic efficiency and poor thermal stability, which hinder the large-scale commercial application for Ni-rich NCM [9,10]. These problems caused by high Ni content are determined by its inherent shortcomings, and the reasons are as follows: (1) the similar ionic radii of Ni^2+^ (0.69 Å) and Li^+^ (0.76 Å) cause Ni^2+^ to tend to occupy the Li^+^ site (i.e., cationic mixing) [11], resulting in irreversible deintercalation of Li^+^ during cycles [12]. In addition, this cation migration will lead to phase transformation and particle cracking [11,13]. The microcracks induced by anisotropic lattice volume contraction or expansion among primary particles during cycling lead to deteriorative cycling performance [14]. (2) Compared with Co–O and Mn–O, the bond length of Ni–O changes more seriously during cycles, resulting in the release of O in NCM particles and phase transformation on the surface [15]. (3) For Ni-rich cathode material, Ni^4+^ may oxidize the electrolyte when the highly reactive material surface is in the delithiated state during cycles, causing the side reaction between the electrode and electrolyte, and resulting in loss of active material and forming a thicker surface film [11], the so-called cathode-electrolyte interphase (CEI) [16]. These undesired reactions will cause more serious electrode polarization and capacity degradation, which has a negative effect on the overall efficiency of the battery [15]. (4) Ni-rich cathode material has a poorer thermal stability in the highly delithiated state [17] and is highly sensitive to air and moisture upon storage and processing [18]. In order to prevent the Li/Ni cation mixing, an excess of lithium is necessary to ensure the achievement of highly ordered Ni-rich material during synthesis. However, the unreacted Li on the surface of NCM particles will react with moisture to form lithium residues, such as Li_2_O/LiOH, Li_2_CO_3_ [7,18,19]. The residues can further react with the electrolyte to form an insulating LiF layer on the surface, which will increase the resistance of the positive electrode [11,20]. In addition, the reaction with the electrolyte also produces gaseous species such as O_2_, CO_2_ and CO [21,22]. This kind of gas generation has a negative impact on the performance and life cycle of LIBs [23,24].

There is no grain boundary intrinsically in the structure of single-crystalline (SC) primary particles, which can effectively alleviate the formation of micro/nanocracks and maintain the structural integrity of Ni-rich single-crystalline lithium nickel cobalt manganese oxides (SC-NCM). The unique structure of single crystalline with the limited electrode/electrolyte-interphase area is also beneficial to alleviate the accumulation of side reaction products and to mitigate undesired phase transformation [25,26]. For the above reasons, Ni-rich SC-NCM primary particles are competitive cathode materials, which can be one of the most promising strategies to enhance structural stability and suppress the interphase parasitic reactions, and hence boost cycling performance and thermal stability [25,27].

Compared to normal polycrystalline NCM with micron-sized secondary particles (PC-NCM), SC-NCM can suppress the side reaction between the electrode and electrolyte, so that the decomposition of the electrolyte and the dissolution of the transition metal are significantly alleviated. As a result, SC-NCM maintains good rectangular-like morphology and mechanical integrity without visible cracks even after long cycles. Only a very thin layer of disordered phase is detected on the surface of the particles, and the $R\bar{3}m$ layered structure is maintained inside the bulk after long cycles at the elevated temperature. Charged electrodes within electrolyte show that SC-NCM electrode has a much higher exothermic temperature and a lower heat generation compared to the PC-NCM electrode. It demonstrates that the unique single-crystalline structure can inhibit the thermal runaway and oxygen release, maintaining structural stability and improving thermal safety [28].

For PC-NCM, ion doping and surface coating with foreign elements are effective research strategies to overcome these drawbacks mentioned above [29,30]. Generally, element doping is widely used to enhance the structural stability of cathode materials, and the stabilization effect is mainly due to the stable M–O bond and the reduction in cation mixing [31,32]. The coating layer can reduce the amount of lithium residuals and inhibit side reactions between organic electrolyte and active materials. In addition, the coating layer can also act as protective film to relieve volume contraction or expansion [33,34]. In terms of element tungsten modification, tungsten can act as either ion doping or surface coating to improve the electrochemical performance of PC-NCM [35,36]. Due to the strong W–O bond, an appropriate amount of W-doping can strengthen the thermal stability of PC-NCM, thereby enhancing electrochemical performance. W-doping can reinforce the stability of the crystal structure of PC-NCM during deep delithiation mainly because W-doping can suppress the release of oxygen. In addition, W-doping can inhibit the lattice expansion and the formation of microcracks during long-term cycling, thereby improving the structure and interface stability. In addition, W-doping can lower the energy barrier of Li^+^ migration and improve Li^+^ diffusion kinetics [35]. Surface coating via tungsten metal compounds can effectively stabilize the interface between the cathode active material and electrolyte, leading to a reduction in parasitic side reactions and the improvement of the electrochemical performance of PC-NCM [36]. However, it has not been systematically investigated whether element tungsten oxide plays the role of ion doping or/and surface coating for PC-NCM. According to previous reports, those results were very different from each other. For instance, tungsten acts as ion doping to increase the stability of materials with the addition of WO_3_ into a coprecipitation reactor during the synthesis of precursors [37,38] or into a furnace during the synthesis of NCM by calcination [35,36]. It is reported that the improvement of the stability for the nickel-rich cathodes is attributed to the migration of W to the Ni site to stabilize oxygen framework [39]. Another report shows that W-doping is most preferred at Co sites to strengthen the W–O bond, thereby stabilizing the crystal structure during deep delithiation [35]. Some results reveal that tungsten oxide plays the role of coating layer rather than doping, to improve electrochemical properties [36,40,41]. Furthermore, the effect of the W element on SC-NCM has not been reported yet. In this paper, the effect of WO_3_ on the surface modification of SC-NCM is researched, and the strategy of surface modification to restrain structural degradation is given.

## 2. Experimental Section

### 2.1. Materials Synthesis

The Ni_0.6_Co_0.2_Mn_0.2_(OH)_2_ precursor was conventional commercial materials. Firstly, the Ni_0.6_Co_0.2_Mn_0.2_(OH)_2_ hydroxide precursors were thoroughly ground and mixed with LiOH·H_2_O (Aladdin, 99.9%, Li:M ratio = 1.06:1). The mixture was then put into muffle furnace and calcined at 950 °C for 10 h in an atmosphere of pure oxygen at an airflow rate of 5 mL min^−1^. After cooling to room temperature at the end of calcination, the obtained sample was ball-milled (FRITSCH PULVERISETTE 7, Idar-Oberstein, Germany) at 200 rpm for one hour to make the particles more dispersed. Finally, Pristine SC-NCM622 and modification products could be obtained by grinding and mixing ball-milled sample and WO_3_ (Aladdin, 99.99%) with mass ratios of 0, 0.5%, 1.0% and 1.5%, and then secondarily calcining at 800 °C for 2 h in argon atmosphere. The four samples were labeled as SC-NCM622, SC-NCM622-0.5%WO_3_, SC-NCM622-1.0%WO3 and SC-NCM622-1.5%WO_3_, respectively.

### 2.2. Materials Characterization

X-ray diffraction (XRD) patterns of the samples were detected by Rigaku-mini Flex 600 X-ray diffractometer (Cu-Kα radiation). Diffraction patterns were obtained in the range of 10°–90° at 0.02° intervals at the scanning speed of 2° min^−1^. Scanning electron microscopy (SEM) (Zeiss Sigma 500, Oberkochen, Germany) was used to observe the morphology, and the images of samples were taken with accelerating voltage of 10.0 kV. High-resolution transmission electron microscopy and energy dispersive spectroscopy (EDS) (HRTEM, FEI Tecnai G2 F20, Portland, OR, USA) were used to further examine microstructure and elemental mapping on the surface of a single particle for the synthesized samples. X-ray photoelectron spectroscopy (XPS, Thermo Fisher Scientific ESCALAB 250Xi system, Waltham, MA, USA) measurements were performed to obtain information about the chemical elements and valence of the samples.

### 2.3. Electrochemical Measurements

Electrochemical performance was tested using CR2032-type coin cells. At room temperature, 80 wt% cathode active materials, 10 wt% acetylene black and 10 wt% PVDF (Polyvinylidene fluoride) were mixed and ground, and then put into NMP (N-methyl-2-pyriolidon) solution under a magnetic stirring for 6–8 h to obtain slurry. The slurry was coated on the aluminum foil and then vacuum-dried at 110 °C for 10 h. After that, the foil was cut into disks with a diameter of 14 mm and active material loading of about 2–5 mg cm^−2^. The batteries were assembled using Li foil as an anode, Celgard 2400 as separator, and 1 m LiPF_6_ in EC:EMC:DMC = 1:1:1 in volume as electrolyte in a glove box filled with Ar.

Charge–discharge tests were performed using CT2001A battery-testing system (Wuhan LAND Electronic Co. Ltd., Wuhan, China) at the range from 0.2 C to 10 C between 2.8–4.4 V. Cyclic voltammetry (CV) was performed at scanning speed of 0.1 mV s^−1^ and potential of 2.5–4.5 V. Electrochemical impedance spectroscopy (EIS) was conducted at 5.0 mV amplitude and a frequency ranged from 0.1 MHz to 0.01 Hz. Both CV and EIS were detected by Gamry Reference 600 electrochemical workstations. All of the above tests were performed at room temperature.

## 3. Results and Discussion

**Figure 1:**
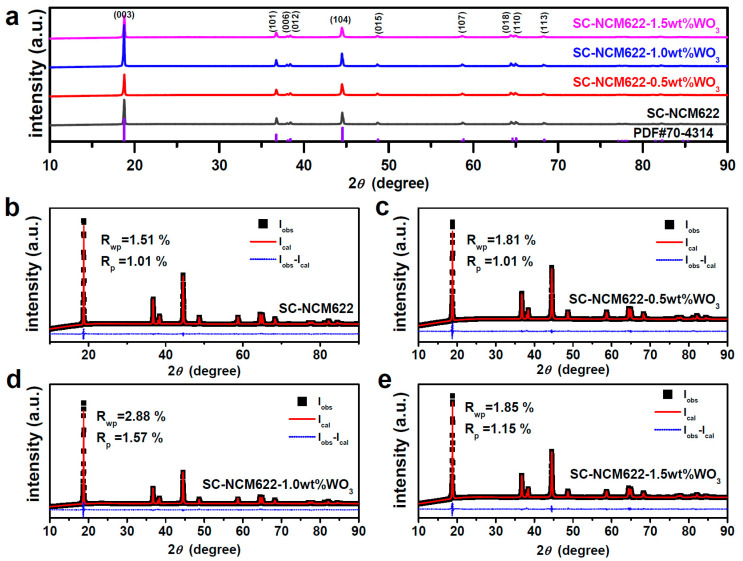
(**a**) X-ray diffraction patterns of the samples, and Rietveld refinement results for (**b**) SC-NCM622, (**c**) SC-NCM622-0.5%WO_3_, (**d**) SC-NCM622-1.0%WO_3_ and (**e**) SC-NCM622-1.5%WO_3_.

XRD patterns of SC-NCM622, SC-NCM622-0.5%WO_3_, SC-NCM622-1.0%WO_3_ and SC-NCM622-1.5%WO_3_ are shown in Figure 1. The diffraction peaks of all the samples show that the materials have layered hexagonal α-NaFeO_2_ structures with an $R\bar{3}m$ space group, which is consistent with PDF card #70-4314 (Figure 1a). No extra diffraction peaks of impurities were detected, indicating that certain amounts of WO_3_ introduced will not change the bulk structure. In addition, the content of the WO_3_ addition was out of the limit of detection. The significant splitting of the (006)/(012) and (018)/(110) peaks manifests that all the samples own well-ordered layered structure [42]. The peak intensity value of I(003)/I(104) reflects the degree of cation mixing in the layered hexagonal structure. It is widely believed that the higher the ratio of I(003)/I(104), the lower the degree of cation mixing, and the layered material suffers a low degree of cationic disorder when the ratio of I(003)/I(104) is beyond 1.20 [43]. The ratio of I(003)/I(104) for all samples is shown in Table 1. The results show that although the addition of WO_3_ into SC-NCM622 may reduce the ratio of I(003)/I(104), an appropriate amount of WO_3_ introduced can significantly increase the value of I(003)/I(104). The ratio of I(003)/I(104) for SC-NCM622-1.0%WO_3_ is much larger than others, indicating that an appropriate amount of WO_3_ introduced can greatly reduce the degree of cation mixing, which is consistent with the results refined by the Rietveld analysis using the GSAS/EXPGUI package [44] (Figure 1b–e), and is also listed in Table 1. The larger R_p_ and R_wp_ for SC-NCM622-1.0%WO_3_ than those of other samples are derived from a more distorted fitting of the (003) peak due to the requirement to cover the whole XRD pattern. Although the R_p_ and R_wp_ values of SC-NCM622-1.0%WO_3_ are the largest, they are also in a reasonable range and do not affect the analysis of the material. Interestingly, SC-NCM622-1.0%WO_3_ manifests Li-rich, rather than Ni on Li sites. This can be attributed to that an appropriate amount of WO_3_ introduced facilitates the formation of layered structure by reducing the crystallization temperature of pristine SC-NCM622. The degree of cationic mixing for SC-NCM622-0.5%WO_3_ and SC-NCM622-1.5%WO_3_ is higher than that of pristine SC-NCM622, indicating that too small or too large an amount of WO_3_ is not conducive to the formation of the layered structure. Too small or too large an amount of added WO_3_ enlarges the lattice constant of a and c. This implies that the unit cell is expanded, indicating that doping is successfully realized after WO_3_ addition resulting from the ionic radius of W^6+^ (0.60 Å) larger than Co^3+^ (0.545 Å), Ni^3+^ (0.56 Å), and Mn^4+^ (0.53 Å) [35,44]. Due to the effect of charge compensation, W doping makes the Ni element more likely to behave as +2 valence, resulting in greater cation mixing, thus the smaller ratio of I(003)/I(104). The increase in lattice parameter c indicates enlarged pathways for the lithiation/delithiation in the samples with WO_3_ introduced, which will be profitable to the transportation of lithium ions and promote rate performance [45]. The lattice constant almost remains unchanged, with the addition of an appropriate amount of WO_3_, indicating that an appropriate amount of WO_3_ acts as coating rather than doping. Even so, the values of a, c and volume have only tiny differences for all samples, indicating that the bulk structures of SC-NCM622 are not damaged by WO_3_ introduced. For all the samples, the c/a ratio is greater than 4.94, which is coordinating with explicitly split (006)/(012) and (018)/(110) peaks, also indicating that the materials own well-ordered layered structures. On the whole, SC-NCM622-1.0%WO_3_ has the optimal ratio of I(003)/I(104) and c/a (Figure S1).

**Figure 2:**
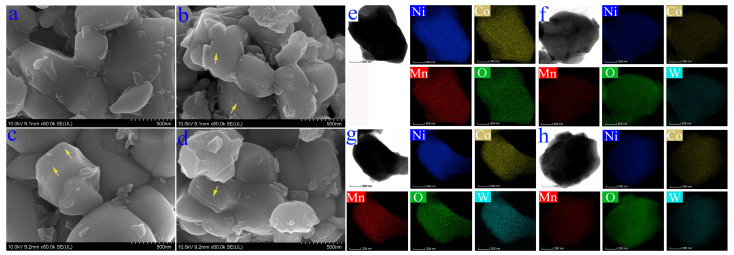
SEM, TEM images and EDS elemental mappings of (**a**,**e**) SC-NCM622, (**b**,**f**) SC-NCM622-0.5%WO_3_, (**c**,**g**) SC-NCM622-1.0%WO_3_ and (**d**,**h**) SC-NCM622-1.5%WO_3_.

SEM shows that the morphology of single-crystalline particles with or without WO_3_ is similar, and they are distributed in bulk shape with different sizes, with particle sizes of 1–2 μm in general (Figure 2). The small particles adhering to the surface and fringe of the large particles are derived from ball milling. The samples with the WO_3_ addition show a more obvious layered structure on the side edges of the particles (see the arrow in Figure 2b–d). TEM and EDS element mapping show that Ni, Co, Mn and O are evenly distributed in the position of the particles, and that the W element is also evenly distributed in the position of the particles for the samples with WO_3_ addition (Figure 2e,f).

**Figure 3:**
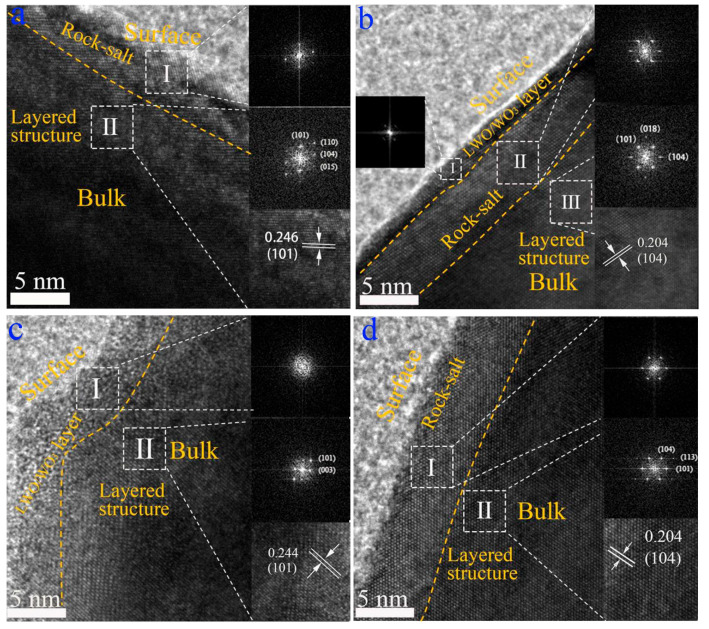
HRTEM images and fast Fourier transformed (FFT) images of selected areas for SC-NCM622, SC-NCM622-0.5%WO_3_, SC-NCM622-1.0%WO_3_, and SC-NCM622-1.5%WO_3_, (**a**) I: Rock-salt phase, II: Layered phase; (**b**) I: LWO/WO_3_ layer, II: Rock-salt phase, III: Layered phase, (**c**) I: LWO/WO_3_ layer, II: Layered phase, (**d**) I: Rock-salt phase, II: Layered phase.

Figure 3 shows HRTEM images and fast Fourier transformed (FFT) images of selected areas for SC-NCM622, SC-NCM622-0.5%WO_3_, SC-NCM622-1.0%WO_3_ and SC-NCM622-1.5%WO_3_, respectively. The outer layer of the particle is the rock-salt phase ($\mathrm{Fm}\bar{3}m$ space group) with a thickness of about 5 nm for the pristine sample. The inside of the particle is the ordered layered phase ($R\bar{3}m$ space group) with an atomic interlayer spacing of 0.246 nm, corresponding to the (101) facets (Figure 3a). The rock-salt phase existing on the surface of the pristine sample is caused by the cation mixing and the formation of the NiO phase after Ni occupies the Li position. Compared with the pristine sample, the SC-NCM622-0.5%WO_3_ sample also has the layered structure and the rock-salt phase. For the SC-NCM622-0.5%WO_3_ sample, the thickness of the rock-salt phase is slightly thicker than that of the pristine sample, which is consistent with the result of greater cation mixing obtained by XRD refinement analysis. The atomic interlayer spacing of the layered phase is 0.204 nm, corresponding to the (104) facets. In addition, the thin LWO/WO_3_ coating layer on the outside of the rock salt phase, with a thickness of 1–2 nm, is detected (Figure 3b). As a protective layer, the coating layer is conducive to isolating the contact between the active material and the electrolyte, increasing the thermal stability and safety, preventing the structural degradation of the active substance during the cycle, so as to increase the cycle stability [36]. Figure 3c shows that the SC-NCM622-1.0%WO_3_ sample has no rock-salt phase, which is consistent with the result of Li-rich rather than cation mixing derived from the XRD refinement analysis. The atomic interlayer spacing of the layered phase is 0.244 nm, which is corresponding to the (101) facets. Outside the layered phase, a coating layer of LWO/WO_3_ is also detected with a thickness of about 5 nm. Compared with the SC-NCM622-0.5%WO_3_ sample, the coating layer of SC-NCM622-1.0%WO_3_ sample is thicker, which can provide more effective protection for active materials. Figure 3d shows that the outer layer of the SC-NCM622-1.5%WO_3_ sample is also the rock-salt phase, and the thickness of the rock-salt phase is greater than 5 nm. The layered phase close to the rock-salt phase is detected. The atomic interlayer spacing of the layered phase is 0.204 nm, corresponding to the (104) facets. Consistent with the results of XRD refinement analysis, the SC-NCM622-1.5%WO_3_ sample has the most significant cation mixing, and therefore has the thickest rock-salt phase layer. Compared with the pristine sample, the crystallization of the rock-salt phase for the SC-NCM622-0.5%WO_3_ and SC-NCM622-1.5%WO_3_ samples is more obvious, so more prominent FFT spots emerge. It should also be noted that there are transition regions between the phase layers for all samples.

**Figure 4:**
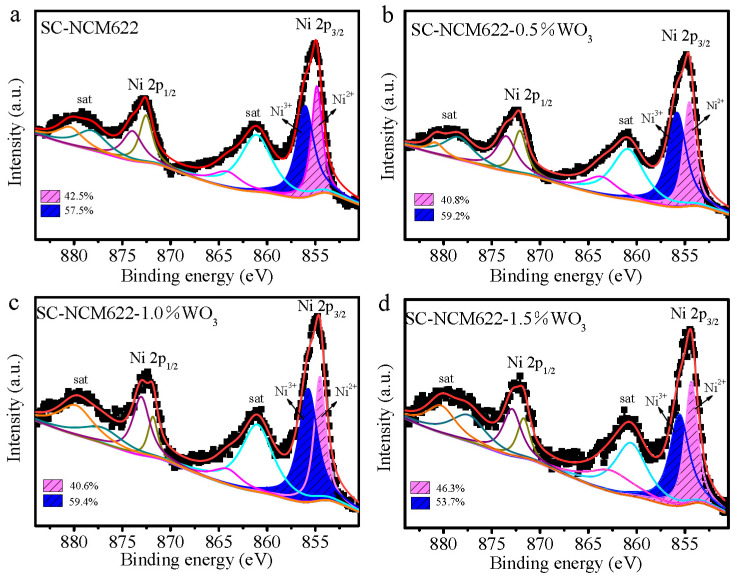
High-resolution XPS spectra and fitting results of Ni 2p for (**a**) SC-NCM622, (**b**) SC-NCM622-0.5%WO_3_, (**c**) SC-NCM622-1.0%WO_3_, and (**d**) SC-NCM622-1.5%WO_3_.

In order to clarify the valence state of each element in the sample, XPS measurements were performed. As shown in Figure 4a for the pristine sample, the peaks at 855.08 eV and 872.68 eV correspond to Ni 2p_3/2_ and Ni 2p_1/2_, and the peaks at 861.58 eV and 879.18 eV are their satellite peaks, respectively. The Ni^3+^ peak at 856.07 eV and the Ni^2+^ peak at 854.86 eV were obtained by fitting the peak of Ni 2p_3/2_, respectively. As seen in Figure 4, The ratios of Ni^2+^/(Ni^2+^ + Ni^3+^) for SC-NCM622, SC-NCM622-0.5%WO_3_, SC-NCM622-1.0%WO_3_ and SC-NCM622-1.5%WO_3_ are 42.5%, 40.8%, 40.6% and 46.3%, respectively. The fitted peak positions and corresponding peak areas are listed in Table S1. The ratio of Ni^2+^/(Ni^2+^ + Ni^3+^) reflects the degree of cation mixing. The larger the ratio is, the higher the degree of cation mixing is [39]. The cation mixing derived from the ratio of Ni^2+^/(Ni^2+^ + Ni^3+^) by fitting XPS is basically consistent with the result obtained by XRD refinement analysis. An appropriate amount of WO_3_ addition reduces the proportion of Ni^2+^, indicating that the amount of added WO_3_ acts as coating rather than doping. On the other hand, excessive WO_3_ addition increases the proportion of Ni^2+^, and hence increases cation mixing, indicating that the addition of excessive WO_3_ leads to the charge-compensation effect of W^6+^ and the achievement of doping [38]. Tungsten doping stabilizes the oxygen element in the lattice by means of the W–O bond and improves the stability of the structure. Moreover, tungsten doping can reduce the energy barrier of Li^+^ migration and improve the diffusion kinetics of Li^+^ [35].

High-resolution XPS spectra and fitting results of Co 2p, Mn 2p, and W 4f are shown in Figure S2. For the pristine sample, the peaks at 780.28 eV and 795.18 eV correspond to Co 2p3/2 and Co 2p1/2, indicating that the valence state of element Co is +3 [36,46]. In addition, the main peaks of Mn 2p3/2 at 642.58 eV and Mn 2p1/2 at 653.98 eV suggest that the valence state of Mn is +4 [36,47]. The peak positions of Co 2p and Mn 2p do not shift significantly after the addition of WO_3_, indicating that the addition of WO_3_ did not change the valence states of the elements Co and Mn [35]. Co 2p3/2 and Mn 2p3/2 are both accompanied by an Ni (LMM) peak [36,48]. W 4f peaks are observed in XPS spectra after the addition of WO_3_. For SC-NCM622-0.5%WO_3_, the W 4f_7/2_ peak at 35.08 eV and the W 4f_5/2_ peak at 37.38 eV indicate that the valence state of W is +6 [21,36,46]. There are also no significant shifts in the W 4f_7/2_ and W 4f_5/2_ peaks for all samples with the addition of WO_3_. LWO/WO_3_ phases are not detected in XRD patterns due to the low content of WO_3_ added in samples. However, there are significant peaks of element W in XPS spectra, demonstrating the realization of LWO/WO_3_ coating and/or doping in the material. It is noted that the high-resolution XPS of the W element cannot distinguish whether the material is coated or doped. Only the high-resolution XPS of the Ni element can provide circumstantial evidence of whether the material is coated or doped. Based on the results of HRTEM and XPS measurements, we cannot determine that the coating layer consists of only one substance of LWO or WO_3_, or both LWO and WO_3_, because WO_3_ can react with the residual LiOH to form LWO compounds during the calcination process. In addition, LWO makes the lithiation/delithiation in Ni-rich cathode materials more reversible by accelerating the migration of Li^+^ [41]. Therefore, WO_3_ and LiOH were mixed and calcined at 400 °C for 2 h and 800 °C for 2 h in an argon atmosphere, respectively. The products were then subjected to XRD tests. The results are shown in Figure S3. After calcination at 400 °C for 2 h, WO_3_ partially reacts with LiOH to form 7Li_2_WO_4_·4H_2_O, and Li_6_WO_6_ forms after WO_3_ reacts with LiOH at 800 °C for 2 h. The XRD results show that WO_3_ and LiOH can easily react with the increase in calcination temperature to form LWO compounds in an argon environment. However, for the samples in which we added WO_3_, the coating layer cannot be distinguished between LWO and WO_3_, since we cannot determine which one is excessive between the addition of WO_3_ and the residual LiOH.

**Figure 5:**
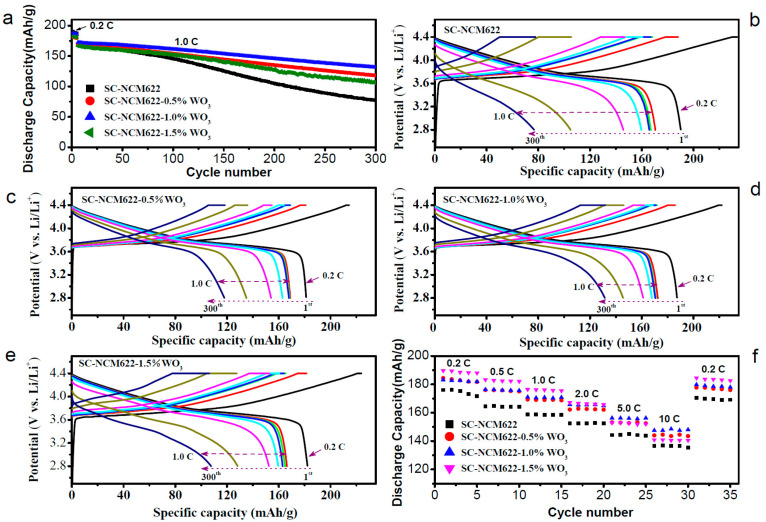
(**a**) Cycle performance of all the samples at 0.2 C rate for the first 5 cycles and 1.0 C rate for subsequent cycles over 2.8–4.4 V. Corresponding charge–discharge curves at the 1st, 6th, 15th, 20th, 50th, 100th, 200th and 300th cycles for (**b**) SC-NCM622, (**c**) SC-NCM622-0.5%WO_3_, (**d**) SC-NCM622-1.0%WO_3_ and (**e**) SC-NCM622-1.5%WO_3_. (**f**) Rate capabilities of all the samples over 2.8–4.4 V.

The cycle performances of coin cells with the four samples as cathodes are shown in Figure 5. The first discharge capacities of SC-NCM622, SC-NCM622-0.5%WO_3_, SC-NCM622-1.0%WO_3_ and SC-NCM622-1.5%WO_3_ at 0.2 C are 190 mAh/g, 181 mAh/g, 187 mAh/g and 182 mAh/g, respectively. The initial capacities of the samples with WO_3_ addition are slightly different. After 5 cycles, the discharge capacities of the first cycle at 1.0 C are 171 mAh/g, 169 mAh/g, 173 mAh/g, and 166 mAh/g, respectively. At 1.0 C, the discharge capacity of SC-NCM622-1.0%WO_3_ is larger than that of the pristine sample. After 300 cycles, the discharge capacities at 1.0 C are 77 mAh/g, 118 mAh/g, 132 mAh/g and 108 mAh/g, and the capacity retention rates are 45.0%, 69.9%, 76.2% and 64.8% for SC-NCM622, SC-NCM622-0.5%WO_3_, SC-NCM622-1.0%WO_3_, and SC-NCM622-1.5%WO_3_, respectively. For the samples with WO_3_ addition, the capacities and capacity-retention rates after a long cycle are greatly improved. Especially for SC-NCM622-1.0%WO_3_, after a long cycle, its discharge capacity increases by 55 mAh/g and the capacity-retention rate increases by 31.2% compared with the pristine sample. The rate performances of other four coin cells based on the four samples as cathodes are shown in Figure 5f. The initial discharge capacities at 0.2 C are 176 mAh/g, 184 mAh/g, 183 mAh/g and 190 mAh/g for SC-NCM622, SC-NCM622-0.5%WO_3_, SC-NCM622-1.0%WO_3_ and SC-NCM622-1.5%WO_3_, respectively. Except for the pristine sample, the initial capacities of other samples are similar to those derived from the cycle performance in Figure 5a. The discharge capacities of the samples with WO_3_ addition are higher than those of the pristine sample at various rates, indicating that both coating and doping can enhance the diffusion ability of the lithium ion and improve the rate performance. At 10.0 C rate, the discharge capacity of SC-NCM622-1.0%WO_3_ is as high as 148 mAh/g, and is 86.3% of that at 1.0 C rate, showing excellent rate performance.

For the SC-NCM622-1.5%WO_3_ sample, W-doping forms a thick layer of rock-salt phase and W–O bonds, which prevent further degradation of the structure during the cycle, thereby enhancing cycle stability. Meanwhile, W-doping increases the channel for Li^+^ diffusion, reduces the energy barrier for Li^+^ diffusion, and improves Li^+^ diffusion kinetics, so as to promote rate performance. For the SC-NCM622-0.5%WO_3_ sample, the layer of rock-salt phase by W-doping is slightly thinner than that in SC-NCM622-1.5%WO_3_ sample. However, it is coated with a thin layer of LWO/WO_3_, which further improves the cycle stability. The synergistic effects of coating and doping make the improvement of Li^+^ diffusion kinetics for SC-NCM622-0.5%WO_3_ sample almost equivalent to that for SC-NCM622-1.5%WO_3_. The SC-NCM622-1.0%WO_3_ sample has no rock-salt phase layer, which can reduce the irreversible delithiation during cycle. In addition, the much thicker LWO/WO_3_ coating layer is more significant in relieving the structural degradation during the cycle and improving the Li^+^ diffusion kinetics, resulting in more excellent cycle stability and rate performance. As shown in Figure 5b–e, the pristine sample shows a more obvious continuous increase in overpotential with the increase in the number of cycles, while the samples with added WO_3_ show more prominent voltage plateaus, indicating that the modified samples have less-resistive CEI and irreversible structural transformation [39]. Figure S4 also gives the similar trends of overpotential and voltage plateau.

**Figure 6:**
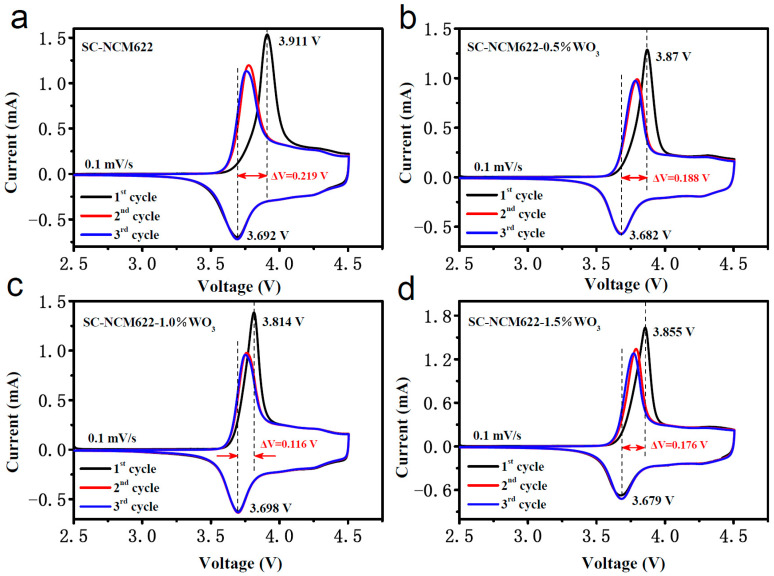
The cyclic voltammetry curves of (**a**) SC-NCM622, (**b**) SC-NCM622-0.5%WO_3_, (**c**) SC-NCM622-1.0%WO_3_ and (**d**) SC-NCM622-1.5%WO_3_ in fresh coin cells.

Figure 6 shows the first three cyclic voltammetry (CV) curves of each sample in fresh coin cells. The peaks appearing in pairs represent the redox reactions of Ni^2+/4+^ and/or Ni^3+^. The oxidation peak is related to the extraction of lithium ions from the bulk crystal lattice, while the reduction peak corresponds to the insertion of lithium ions into the bulk crystal lattice [49]. The potential difference of the redox peak describes the reversibility of the electrochemical process, reflecting the degree of polarization [50,51]. The larger the difference is, the greater the polarization is. The oxidation peaks and reduction peaks of the four samples are 3.911 and 3.692 V; 3.87 and 3.682 V; 3.814 and 3.698 V; and 3.855 and 3.679 V, and the difference between the redox peaks are 0.219, 0.188, 0.116 and 0.176 V, respectively. The results show that both coating and doping reduce polarization. Consistent with the results of XRD and HRTEM, the SC-NCM622-1.0%WO_3_ sample has the minimum polarization, so it shows the best electrochemical performance. After 10 cycles of charged–discharged at 1.0 C, another three cyclic voltammetry tests are carried out for the samples, and the results are shown in Figure S5. The potential differences between the oxidation peak and the reduction peak of SC-NCM622, SC-NCM622-0.5%WO_3_, SC-NCM622-1.0%WO_3_ and SC-NCM622-1.5%WO_3_ are 0.157, 0.095, 0.098 and 0.131 V, respectively. Compared with the pristine sample, the redox peaks of SC-NCM622-0.5%WO_3_, SC-NCM622-1.0%WO_3_ and SC-NCM622-1.5%WO_3_ are more symmetrical and the potential differences are smaller, suggesting that sole doping, sole coating, or both doping and coating can reduce the polarization of the sample and improve electrochemical reversibility during the lithiation/delithiation process, and hence improve electrochemical performance.

**Figure 7:**
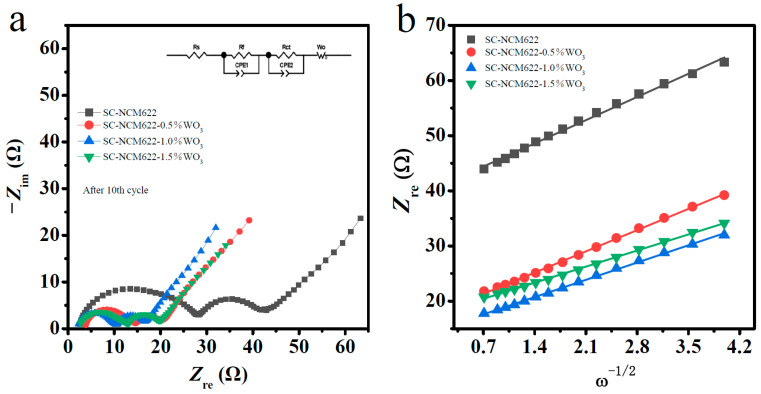
(**a**) Nyquist plots at charge state of 4.4 V after 10th cycle; (**b**) the relationships between Z_re_ and $\omega^{-1/2}$ for SC-NCM622, SC-NCM622-0.5%WO_3_, SC-NCM622-1.0%WO_3_ and SC-NCM622-1.5%WO_3_.

To further study the kinetic process of the materials, electrochemical impedance spectroscopy (EIS) tests were carried out. Figure 7a shows Nyquist plots and equivalent circuit diagrams for EIS results fitting of all the samples charged to 4.4 V after the 10th cycle at room temperature. The Nyquist plots of all samples are composed of two suppressed semicircles in the high-frequency range and the middle-frequency range, and one oblique line in the low-frequency range. Among them, R_s_ is related to electrolyte resistance, R_f_ in the high-frequency region is the resistance of the solid electrolyte membrane, R_ct_ in the middle-frequency region represents the charge transfer resistance, and the slope of the straight line formed in the low-frequency region is related to material transfer Warburg coefficient [36,52,53]. According to the equivalent circuit diagram, the fitted parameters are obtained with Zview software and are listed in Table 2. Compared with the pristine sample, SC-NCM622-0.5%WO_3_, SC-NCM622-1.0%WO_3_ and SC-NCM622-1.5%WO_3_ show smaller R_f_ and R_ct_. The σ coefficient is obtained from the linear fitting of Z_re_ versus $\omega^{-1/2}$ in Figure 7b. Based on the σ coefficient, the lithium-ion diffusion coefficients D_Li_^+^ of SC-NCM622, SC-NCM622-0.5%WO_3_, SC-NCM622-1.0%WO_3_ and SC-NCM622-1.5%WO_3_ are 1.14 × 10^−15^ cm^2^/s, 1.36 × 10^−15^ cm^2^/s, 2.06 × 10^−15^ cm^2^/s and 2.37 × 10^−15^ cm^2^/s, respectively. For the WO_3_-modified samples, the lithium-ion diffusion coefficients increase. As a whole, the kinetic activity of the SC-NCM622-1.0%WO_3_ sample is optimum.

**Figure 8:**
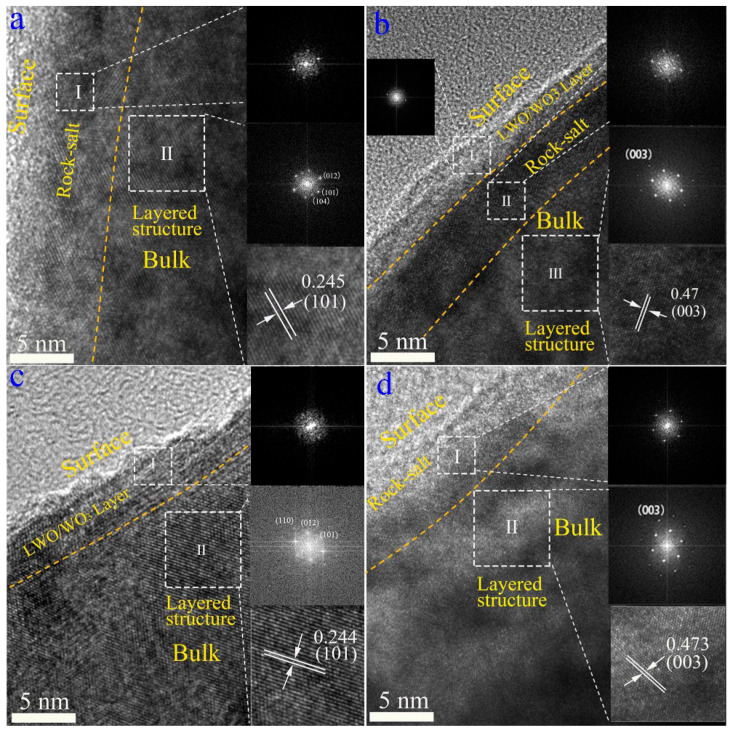
HRTEM images and fast Fourier transformed (FFT) images of selected areas for SC-NCM622, SC-NCM622-0.5%WO_3_, SC-NCM622-1.0%WO_3_, and SC-NCM622-1.5%WO_3_ after 300 cycles. (**a**) I: Rock-salt phase, II: Layered phase; (**b**) I: LWO/WO_3_ layer, II: Rock-salt phase, III: Layered phase, (**c**) I: LWO/WO_3_ layer, II: Layered phase, (**d**) I: Rock-salt phase, II: Layered phase.

To further verify the reasons for improving the electrochemical performance of SC-NCM622 with WO_3_ addition, HRTEM was again used to detect the active materials after they were cycled 300 times at 1.0 C and scraped from electrodes, as shown in Figure 8. The FFT images of selected areas of each sample are also given. For the pristine sample, the outer rock-salt phase layer is about 8.0 nm, much thicker than that before cycling. Contrary to maintaining stability, the transition metal elements can continuously migrate to the lithium layer and cause a phase change on the surface of the material, so that the rock-salt layer becomes thicker and thicker along with the increase in cycling times, which is not conducive to the maintenance of the inner layered structure. For the samples initially forming thicker rock-salt phases by doping (SC-NCM622-0.5%WO_3_ and SC-NCM622-1.5%WO_3_), the further transition of layered phase to rock-salt phase during cycling is alleviated, because doping leads to a more stable surface rock-salt phase. After cycling 300 times, the coating layers for the SC-NCM622-0.5%WO_3_ and SC-NCM622-1.0%WO_3_ samples and the rock-salt phase layers for the SC-NCM622-0.5%WO_3_ and SC-NCM622-1.5%WO_3_ samples change slightly. For the samples with WO_3_ addition, the more steady coating layer and/or rock-salt phase layer can inhibit the inner layered structure collapse, preventing the fragmentation of single-crystalline particles to a certain extent, resulting in better stability [14]. For the SC-NCM622-1.0%WO_3_ sample, the thicker coating layer and the lack of rock-salt phase layer give the SC primary particle a more stable layered structure. This further explains the mechanism of the SC-NCM622-1.0%WO_3_ sample having the best cycle stability after 300 cycles.

## 4. Conclusions

The SC LiNi_0.6_Co_0.2_Mn_0.2_O_2_ with modification of WO_3_ is obtained by a simple two-step calcination method, and the Ni-rich SC-NCM cathode material can be individually coated, individually doped, and both coated and doped by adjusting the amount of WO_3_. WO_3_ modification effectively improves the structural stability of the Ni-rich SC-NCM cathode material, inhibits the electrochemical polarization of the material, reduces the resistance, and increases the lithium-ion diffusion coefficient, hence significantly improves electrochemical performance. Among them, the individually coated sample SC-NCM622-1.0%WO_3_ has the most excellent electrochemical performance since the disappearance of the rock-salt phase layer eliminates the mixing of cations and reduces the irreversible delithiation and phase transition during the cycle, and the effective coating enhances the structural stability. As a result, the discharge capacity at the 10.0 C rate is 86.3% of that at 1.0 C rate. Moreover, after 300 cycles at 1.0 C, the discharge capacity is maintained 132 mAh/g and the capacity retention rate is 76.2%, which is 31.2% higher than that of the pristine sample. This study reveals the effect of WO_3_ on the surface modification of SC-NCM and the mechanism of performance improvement, which are of great significance for improving the electrochemical performance of SC-NCM cathode materials. Meanwhile, this study opens a window for in-depth discussion on the mechanism of the content change of modifiers modulating single doping, single coating, and both coating and doping, which is a target worthy of further investigation.

## Figures and Tables

**Table 1:** Crystallographic data from Rietveld refinement for prepared LiNi_0.6_Co_0.2_Mn_0.2_O_2_ without/with WO_3_ modification.

Sample	a (Å)	c (Å)	c/a	V (Å^3^)	Ni% on Li Sites	R_p_ (%)	R_wp_ (%)	I_(003)_/I_(104)_
SC-NCM622	2.868	14.213	4.955	101.264	1.03	1.01	1.51	1.24
SC-NCM622-0.5%WO_3_	2.872	14.224	4.953	101.590	2.94	1.01	1.81	1.07
SC-NCM622-1.0%WO_3_	2.868	14.211	4.954	101.259	Li-rich 0.87	1.57	2.88	1.69
SC-NCM622-1.5%WO_3_	2.872	14.221	4.952	101.565	3.04	1.15	1.85	1.00

**Table 2:** The kinetic parameters of SC-NCM622, SC-NCM622-0.5%WO_3_, SC-NCM622-1.0%WO_3_ and SC-NCM622-1.5%WO_3_.

Samples	Cycles	R_s_ (ohm)	R_f_ (ohm)	R_ct_ (ohm)	CPE1 (mF)	CPE2 (mF)
SC-NCM622	10th	2.097	24.13	15.01	1.49 × 10^−2^	4.437
SC-NCM622-0.5%WO_3_	10th	3.497	10.23	6.087	9.587 × 10^−3^	5.882
SC-NCM622-1.0%WO_3_	10th	1.887	8.207	5.849	6.933 × 10^−3^	3.400
SC-NCM622-1.5%WO_3_	10th	2.102	10.48	6.359	2.468 × 10^−2^	4.800

## Data Availability

Data are contained within the article.

## Supplementary Materials

The following figures are available online at https://www.mdpi.com/article/10.3390/nano12081324/s1. Figure S1: The value of c/a and *I*(003)/*I*(104) as functions of the amount of WO_3_ addition. Figure S2: High resolution XPS spectra and fitting results of (a) Co 2p, (b) Mn 2p, and (c) W 4f for SC-NCM622, SC-NCM622-0.5%WO_3_, SC-NCM622-1.0%WO_3_, and SC-NCM622-1.5%WO_3_. Figure S3: XRD results of WO_3_ mixed with LiOH and calcined at (a) 400 °C and (b) 800 °C in argon atmosphere. Figure S4: The charge-discharge curves of the 1st cycle at 0.2 C, 0.5 C, 1.0 C, 2.0 C, 5.0 C, and 10.0 C for (a) SC-NCM622, (b) SC-NCM622-0.5%WO_3_, (c) SC-NCM622-1.0%WO_3_, and (d) SC-NCM622-1.5%WO_3_ derived from rate performances. Figure S5: The cyclic voltammetry curves after 10 cycles at 1.0 C of (a) SC-NCM622, (b) SC-NCM622-0.5%WO_3_, (c) SC-NCM622-1.0%WO_3_, and (d) SC-NCM622-1.5%WO_3_. Table S1: Fitting data of Ni 2p3/2 derived from XPS spectra for the pristine sample and SC-NCM622 modified with WO_3_.
