# Supplementary material for: Multi-Role Surface Modification of Single-Crystalline Nickel-Rich Lithium Nickel Cobalt Manganese Oxides Cathodes with WO3 to Improve Performance for Lithium-Ion Batteries
Source: Nanomaterials (Basel). 2022 Apr 12;12(8):1324. doi: 10.3390/nano12081324 (PMC9026308; doi:10.3390/nano12081324)
Supplement: Supplementary file 1 [file nanomaterials-12-01324-s001.zip › nanomaterials-1665301-supplementary.pdf]

nanomaterials

Supplementary Materials

Multi-Role Surface Modification of Single-Crystalline Nickel-Rich Lithium Nickel Cobalt Manganese Oxides Cathodes with WO_3 to Improve Performance for Lithium-Ion Batteries

Limin Ou, Shengheng Nong, Ruoxi Yang, Yaoying Li, Jinrong Tao, Pan Zhang, Haifu Huang, Xianqing Liang, Zhiqiang Lan, Haizhen Liu, Dan Huang, Jin Guo and Wenzheng Zhou *

Guangxi Novel Battery Materials Research Center of Engineering Technology, Guangxi Colleges and Universities Key Laboratory of Novel Energy Materials and Related Technology, Guangxi Key Laboratory of Processing for Non-ferrous Metallic and Featured Materials, School of Physical Science and Technology, Guangxi University, Nanning 530004, China; 1907301067@st.gxu.edu.cn (L.O.); 2007301111@st.gxu.edu.cn (S.N.); 2107301160@st.gxu.edu.cn (R.Y.); 2107301062@st.gxu.edu.cn (Y.L.); 1812110135@st.gxu.edu.cn (J.T.); 1812110145@st.gxu.edu.cn (P.Z.); huanghf@gxu.edu.cn (H.H.); lxq@gxu.edu.cn (X.L.); l_zq1100@163.com (Z.L.); liuhz@gxu.edu.cn (H.L.); danhuang@gxu.edu.cn (D.H.); guojin@gxu.edu.cn (J.G.)

* Correspondence: wzzhou@gxu.edu.cn

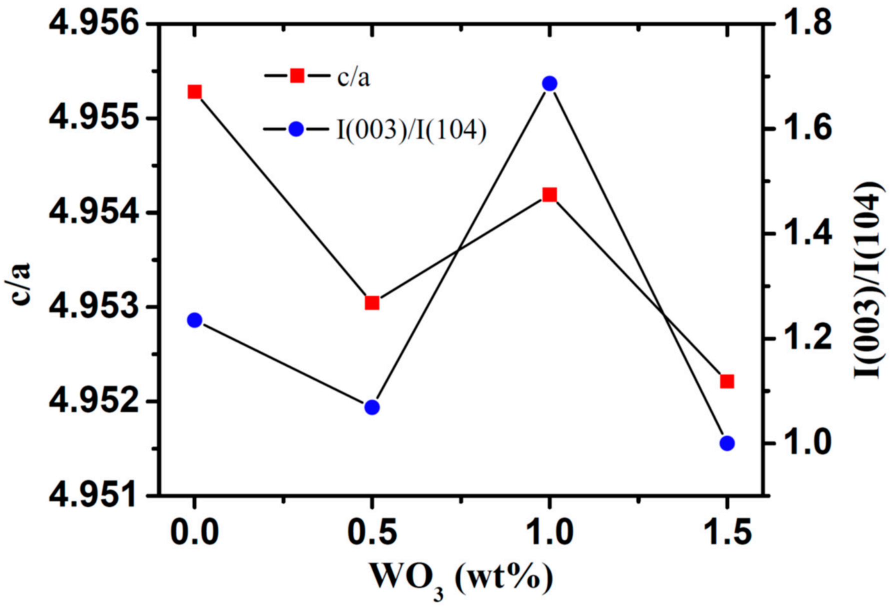

Figure S1. The value of c/a and I(003)/I(104) as functions of the amount of WO_3 addition.

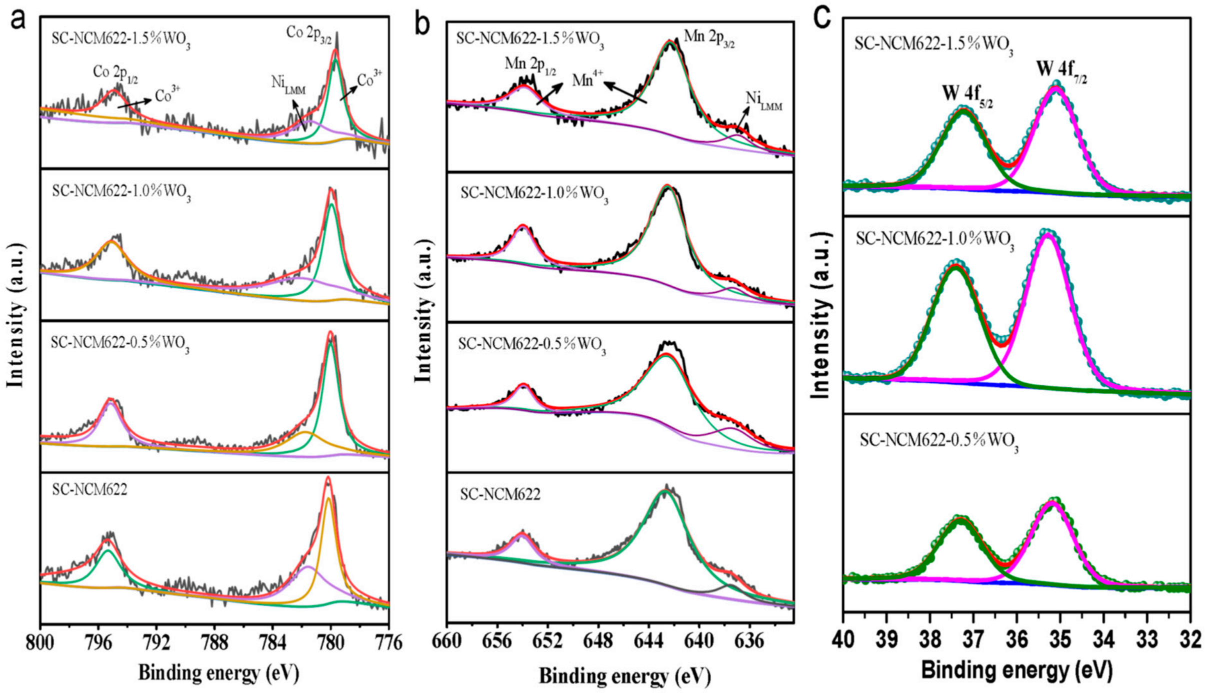

Figure S2. High resolution XPS spectra and fitting results of (a) Co 2p, (b) Mn 2p, and (c) W 4f for SC-NCM622, SC-NCM622-0.5%WO_3, SC-NCM622-1.0%WO_3, and SC-NCM622-1.5%WO_3.

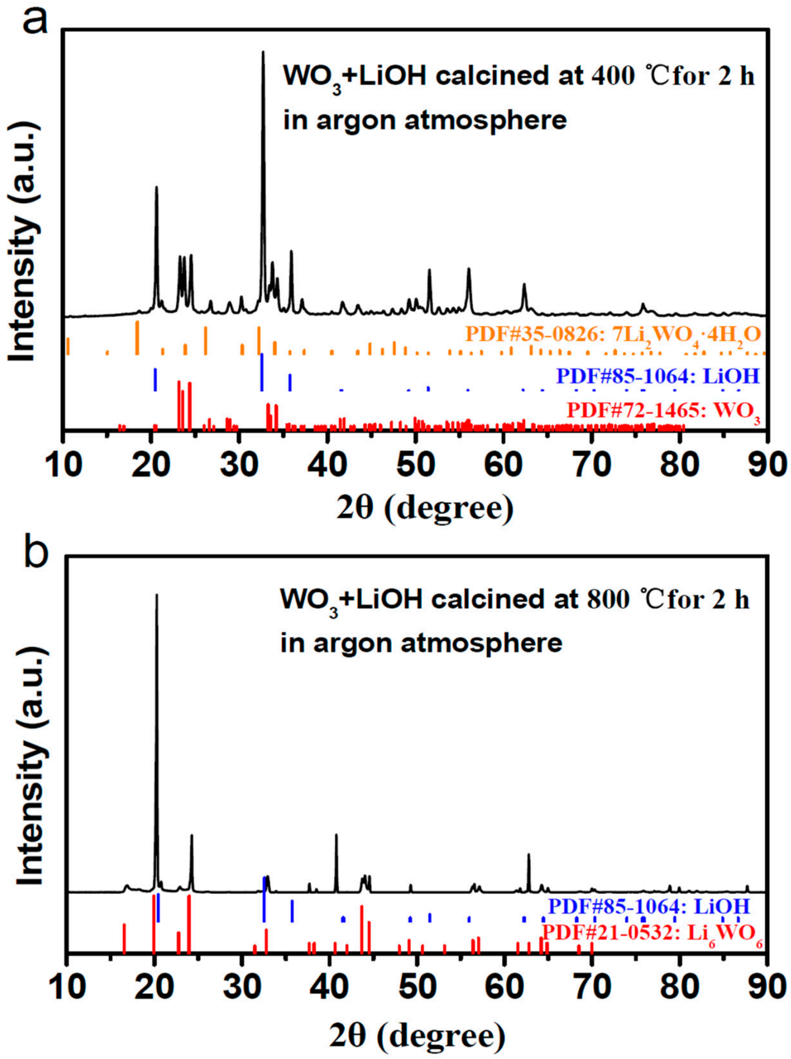

Figure S3. XRD results of WO_3 mixed with LiOH and calcined at (a) 400 ℃ and (b) 800 ℃ in argon atmosphere.

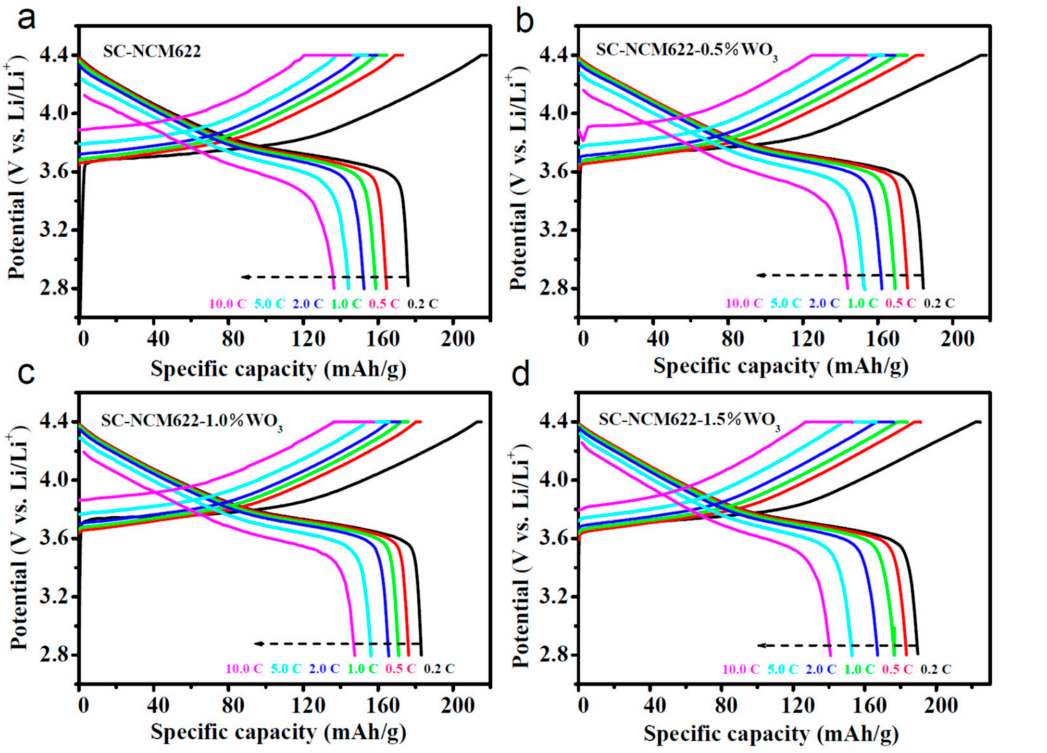

Figure S4. The charge-discharge curves of the 1st cycle at 0.2 C, 0.5 C, 1.0 C, 2.0 C, 5.0 C, and 10.0 C for (a) SC-NCM622, (b) SC-NCM622-0.5%WO_3, (c) SC-NCM622-1.0%WO_3, and (d) SC-NCM622-1.5%WO_3 derived from rate performances.

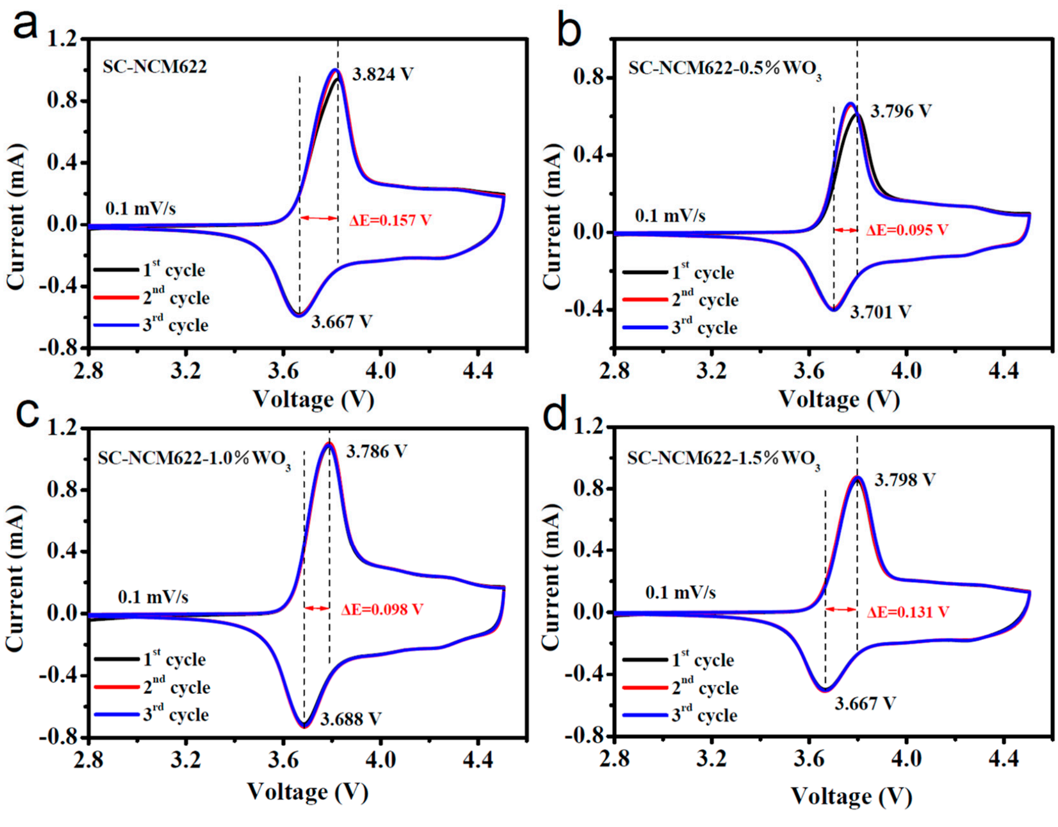

Figure S5. The cyclic voltammetry curves after 10 cycles at 1.0 C of (a) SC-NCM622, (b) SC-NCM622-0.5%WO_3, (c) SC-NCM622-1.0%WO_3, and (d) SC-NCM622-1.5%WO_3.

Table S1. Fitting data of Ni 2p3/2 derived from XPS spectra for the pristine sample and SC-NCM622 modified with WO_3.

Sample	Ni^{2+}		Ni^{3+}		Ni^{2+}/(Ni^{2+}+ Ni^{3+}) (%)
	Peak (eV)	Area	Peak (eV)	Area	
**SC-NCM622**	854.86	27856.35	856.07	37750.74	42.5
SC-NCM622-0.5%WO_3	854.54	43739.03	855.83	63406.86	40.8
SC-NCM622-1.0%WO_3	854.53	21730.31	855.75	31739.24	40.6
SC-NCM622-1.5%WO_3	854.34	14700.67	855.58	17081.56	46.3
